# Low Healthy Diet Self-Efficacy and Intentions Associated with High Sweet Snacks and Sugar Sweetened Beverages Consumption among African American Adolescents Recruited from Low-Income Neighborhoods in Baltimore

**DOI:** 10.3390/nu13124516

**Published:** 2021-12-17

**Authors:** Isna A. Fajarini, Mika Matsuzaki, Cara F. Ruggiero, Caroline R. Wensel, Sangwon Chung, Laura Hopkins, Lisa Poirier, Uriyoán Colón-Ramos, Joel Gittelsohn

**Affiliations:** 1 Center for Human Nutrition, Bloomberg School of Public Health, Johns Hopkins University, Baltimore, MD 21205, USA; mmatsuz2@jhu.edu (M.M.); lpoirie4@jhu.edu (L.P.); jgittel1@jhu.edu (J.G.); 2 Center for Childhood Obesity Research, Department of Nutritional Sciences, The Pennsylvania State University, State College, PA 16801, USA; cfr8@psu.edu; 3 Department of Medicine, School of Medicine, Johns Hopkins University, Baltimore, MD 21205, USA; cwensel1@jhmi.edu; 4 Korea Food Research Institute, Jeonju-si 55365, Jeollabuk-do, Korea; schung@kfri.re.kr; 5 Department of Public Health and Prevention Sciences, College of Education and Health Sciences, Baldwin Wallace University, 328D Malicky Center, 275 Eastland Road, Berea, OH 44017, USA; lhopkins@bw.edu; 6 Milken Institute School of Public Health, George Washington University, Washington, DC 20052, USA; uriyoan@gwu.edu

**Keywords:** adolescent, African American, food intentions, self-efficacy, sweet snacks, sugar sweetened beverages

## Abstract

Psychosocial factors may influence consumption patterns of sweet snacks and sugar sweetened beverages (SSB), which are potential risk factors for obesity among African American (AA) adolescents. We used multivariable linear and logistic regression models to examine cross-sectional associations among psychosocial factors, sweet snacks and SSB consumption, and BMI z-scores in 437 AA adolescents aged 9–14 years living in low-income neighborhoods in Baltimore City, U.S.A. Mean caloric intake from sugar was 130.64 ± 88.37 kcal. Higher sweet snacks consumption was significantly associated with lower self-efficacy (adjusted Odds Ratio (aOR) = 0.81; 95% CI = 0.71 to 0.93) and lower food intentions scores (0.43; 0.30 to 0.61). Higher SSB consumption was associated with lower outcome expectancies (aOR = 0.98; 95% CI = 0.96–0.99), lower self-efficacy (0.98; 0.96 to 0.99), and lower food intentions (0.91; 0.87 to 0.95). No significant association was found between SSB and sweet snacks consumption and weight status. Psychosocial factors may play a role in sugar consumption behaviors among AA adolescents in low-income neighborhoods. Further studies are needed to improve our understanding of causal mechanisms of this association.

## 1. Introduction

The burden of obesity disproportionately affects African American (AA) adolescents in the U.S. The prevalence among AA adolescents has continued to increase over the last four decades, consistently higher than their non-Hispanic white counterparts [1,2]. According to the National Center of Health Statistics (NCHS) data in 2018, the number of AA boys with obesity was two times higher than white boys, and three times higher for girls [2]. This issue raises a substantial concern in public health, as obesity in adolescents is closely lined with lower self-esteem and lower school performance as well as higher risk of cardiovascular diseases and type 2 diabetes in adulthood [3,4,5].

Sugar sweetened beverages (SSB) and sweet snacks have long been recognized as some of the potential contributors to youth obesity. A number of studies have suggested that the consumption of sugar and SSB has decreased in the overall population since the late 1990s [6]. However, this decrease is not uniform across racial/ethnic groups or weight status; for instance, declines in the number of calories from SSB were observed between 2003–2006 and 2007–2010 among AA adolescents at healthy weight, but not with overweight/obesity [7]. In comparison to other racial or ethnic groups, studies have shown higher consumption of sugary products among African Americans [8,9,10]. Among schoolchildren, AA consume more non-soda SSB (e.g., lemonade, sports drinks) and less low-fat milk than their non-Hispanic white counterparts [8]. Given increased autonomous decisions on food choices [11], as well as the potential formation of long-lasting dietary habits during adolescence years [12,13], this period presents a crucial window of opportunity to implement nutritional interventions to promote healthy eating behaviors.

The Social Cognitive Theory (SCT) by Albert Bandura provides a theoretical framework for explaining and predicting behavioral changes with six core constructs [14]. Some studies have found self-efficacy as the key construct associated with sweet snacks and SSB consumption [15]. However, this association may be context dependent as variable results have been observed across studies [16], which warrants further investigation in specific subgroups at high risk of high consumption of sugary products. Other psychosocial factors, such as knowledge and attitudes, may also be associated with, and perhaps even predictive of, dietary behaviors [17,18]. A previous study found that intervention to improve health knowledge was associated with lower sugary intake among racial/ethnic minority adolescents from low-income neighborhoods in Baltimore. The relationship between knowledge and sweet snacks consumption may be mediated by psychosocial factors such as attitude and intention. The same study in Baltimore found that among AA adolescents, limited knowledge related to sugar is associated with a more positive attitude towards sweet snacks and greater intention to purchase sweet snacks [19]. Further, adolescents who perceived SSB as “safe” or as not causing harm to the body had a higher risk to consume energy drinks more often [20].

Consideration to various psychosocial factors may improve the effectiveness of interventions to change dietary behaviors among adolescents. However, the relationship between these factors and dietary behaviors among AA adolescents from low-income neighborhoods is currently understudied. Our study aimed to strengthen the evidence about which potentially modifiable psychosocial factors may affect sugar consumption among AA adolescents with the following research questions:

1. What are the patterns of sweet snacks and SSB consumption in a sample of low-income urban AA adolescents?
2. What is the relationship between AA adolescents’ psychosocial factors (healthy diet knowledge, outcome expectancies, self-efficacy, intention) and their sweet snacks and SSB consumption?
3. What is the relationship between sweet snacks and SSB consumption with overweight and obesity among AA adolescents living in low-income neighborhoods?

## 2. Materials and Methods

### 2.1. Study Design and Sample

The study used the baseline data from a group randomized controlled intervention trial named B’more Healthy Communities for Kids (BHCK) conducted in Baltimore, Maryland, USA [21]. BHCK was a multilevel and multicomponent intervention targeting low-income AA youth aged 9–15 to prevent obesity by intervening at multiple levels of the food and social environments by increasing access, demand, and consumption of healthier foods. Low-income areas in Baltimore City were selected as the study location due to its limited availability of healthy foods and food source in general [22]. The city had many corner stores, carry-out restaurants, and fast-food restaurants, the majority of which stock primarily items high in added sugar items [23]. In 2007, two-thirds of Baltimore city adult residents were either overweight or obese [24]. Baseline data were collected in two waves: July 2013 to June 2014 (Wave 1), and August 2015 to January 2016 (Wave 2)—data from both waves were combined and presented in this study. The detailed explanation of the intervention has been previously published [25].

Study participants were adolescents aged 9–14 and were actively recruited from 28 low-income, predominantly AA neighborhoods categorized as food deserts [25]. Participants were recruited from recreation centers, libraries, swimming pools, grocery stores, and back-to-school events. They were then screened for eligibility criteria, including (1) residing within a mile and a half radius of the neighborhood recreation center and (2) having no intentions of moving within the next two years. Data were collected by data collectors trained on enrollment, consenting process, general questionnaire techniques, and anthropometric measures. Adolescents who did not identify themselves as AA, had no measured body weight and height, or were underweight (*n* = 2) were excluded from the analyses. A total of 437 adolescents were analyzed in this study.

### 2.2. Measures

#### 2.2.1. Sweet snacks and SSB Intake

Dietary intake was assessed using The Block Kids 2004 Food Frequency Questionnaire (BKFFQ)—a semi-quantitative FFQ, validated in adolescents [26]. The BKFFQ contains a list of foods identified by NHANES II as commonly consumed by adolescents. The consumption data were then analyzed by NutritionQuest (Berkeley, CA, USA). NutritionQuest calculates sweet snacks consumption as a percentage of total kcal from sweets, which included sweet and grain-based desserts (i.e., sweet cereal, ice cream, cookies, donuts, cake, chocolate candy, pudding flan). SSB include consumption of regular soda, sports drinks, sweetened iced tea, etc.) expressed in total kcal and grams consumed per day.

#### 2.2.2. Psychosocial Factors

The study assessed various psychosocial factors through a series of scales that were developed and assessed previously, as described in detail elsewhere [21]. In brief, the following psychosocial factors were assessed for this study:

Healthy diet knowledge was assessed using 14 question items by asking adolescents which food is a better option for healthy eating, for example, “which snacks has less sugar?”. There were four response options for each question, each correct answer was scored as 1, and all other answers were scored as 0. The total score ranging from 3 to 14, with a mean ± SD of 9.1 ± 2.5 (α = 0.63) [27].

Healthy diet outcome expectancies were measured using 11 question items regarding the expected health outcome of eating and drinking foods and beverages, for example, “I would lose weight if I drank diet soda instead of regular soda”. The answer choices included “true”, “mostly true”, “mostly false”, “false”, or “don’t know”. Two points were given for the “true” response, 1 for “mostly true”. The total score ranging from 1 to 22, with a mean ± SD of 15.8 ± 3.65 (α = 0.61) [27].

Healthy diet self-efficacy was assessed by 12 items assessing how easy or how difficult it would be for adolescents to perform healthier eating behaviors, for example, “I can drink sugar-free drinks such as Crystal Light instead of fruit punch”. The answers were scored from 0 (the lowest self-efficacy) to 3 (the highest self-efficacy). The total scores ranged from 7 to 36, with a mean ± SD of 28.4 ± 2.0 (α = 0.69) [27].

Healthy diet food intentions were measured by asking children what they would choose to eat, for example, “If you wanted a snack, which would you choose?”. There were three answer choices; the healthy choice was scored as 1 and 0 otherwise. The total scores ranging from 0 to 11, with a mean ± SD of 3.6 ± 2.0, and weak reliability (α = 0.43) [27].

#### 2.2.3. Weight Status

Adolescent height and weight were measured using a Seca 213 Portable Measuring Rod Stadiometer and a Tanita BF697W Duo Scale. Measurements were taken in duplicate; a third measurement was taken if the difference in the first two measurements was greater than 0.25 in or 0.2 lb. The results were then averaged. BMI was calculated (kg/m^2^) as well as BMI-for-age percentile using the Center for Disease Control and Protection (CDC) growth charts, then categorized into normal, overweight, and obese. Adolescents were classified as having overweight if the BMI-for-age between ≥85th and <95th percentile and obesity if ≥95th percentile [28].

### 2.3. Statistical Analysis

Data were analyzed using STATA/IC 16.1. An independent *t*-test was used to assess the difference in sweet snacks and SSB consumption based on respondents’ sociodemographic characteristics that were continuous variables, and a chi-square test was used to assess categorical variables. Statistical significance was set at *p*-value < 0.05.

Multivariable linear regressions were used after linearity, independency, normality, and equal variance assumptions were met. These models assessed the association between (1) each psychosocial factor as an independent variable and sweet snacks consumption (in percentage of total kcal and grams) as the dependent variable; and (2) each psychosocial factor and SSB consumption (in total daily kcal). Both models were adjusted for covariates, including adolescent age and sex, caregiver age and sex, household income, intervention zone, housing arrangement, and food assistance (SNAP) participation; these confounding factors were identified from previous published paper using BHCK dataset and found to be associated with adolescents’ psychosocial factors and their consumption [29,30]. Confounding factors were then corrected by adding all covariates simultaneously to each model during the analysis.

Logistic regressions were used to assess the association between (1) sweet snacks consumption (in percentage and grams) as independent variables and adolescents’ weight status as dependent variable and (2) SSB consumption (in percentage and grams) and adolescents’ weight status. Both models were adjusted for covariates, adolescent age and sex, total daily kcal intake, and household income; these confounding factors were identified from previous published paper using BHCK dataset and found to be associated with adolescents’ psychosocial factors and their consumption [29,30]. Confounding factors were then corrected by adding all covariates simultaneously to each model during the analysis.

## 3. Results

### 3.1. Sample Characteristics and Pattern of Sweet Snacks and SSB Consumption

Adolescents’ average total daily caloric intake was 1735.96 ± 1063.82 kcal, the average percentage of calories from sweet snacks was 14.93 ± 7.28%, and the average daily caloric intake from SSB was 157.93 ± 157.98 kcal. In total, 22.48% of adolescents were overweight, and 26.15% were obese. A total of 87.61% of adolescents’ caregivers were female, 39.08% were high school graduates, and 36.70% had annual household income > USD 30,000 (Table 1).

A significant relationship between SSB consumption based on adolescents’ age was found in multivariable models; younger adolescents aged 9–12 years consumed significantly less SSB compared to older adolescents aged 13–15 years. Moreover, adolescents with caregivers that were college educated consumed more sweet snacks than adolescents with a caregiver with less than high school education (Table 2).

### 3.2. Relationship between AA Adolescents’ Psychosocial Factors and Sweet Snacks and SSB Consumptiom

Adolescents with a higher score in healthy diet outcome expectancies tended to consume less SSB, but not sweet snacks (Table 3). Further, a higher healthy diet self-efficacy score was also associated with less sweet snacks and SSB consumption. However, the association was non-significant after adjustment for adolescents’ and caregivers’ age and sex, caregivers’ education level, household income, and SNAP participation. In addition, adolescents with a higher score in healthy diet food intentions consumed fewer sweet snacks and SSB consumption. In contrast, there was no significant association between healthy diet knowledge and sweet snacks and SSB consumption, before or after adjustment.

### 3.3. Relationship of Sweet Snacks and SSB Consumption with Overweight and Obesity

We found no significant difference in the risk overweight and obesity based on sweet snacks and SSB consumption (Table 4), even after adjusting for adolescents’ age, sex, and total daily caloric intake.

## 4. Discussion

To our knowledge, this is the first paper to assess the psychosocial factors associated with sweet snacks and SSB consumption in a sample of AA adolescents. We found higher sweet snacks consumption but lower SSB consumption among older adolescents than their younger counterparts. Higher healthy food self-efficacy and higher healthy food intentions were associated with a lower consumption of sweet snacks and SSB in this sample. Higher outcome expectancies were associated with lower SSB consumption before adjusting for adolescents’ and caregivers’ characteristics. However, we found that knowledge was not associated with the consumption of any of the foods and beverages assessed. Further, the consumption of sweet snacks and SSB was not significantly associated with adolescents’ risk of overweight and obesity before and after adjusting for their age, sex, and total daily caloric intake.

Data on grams and caloric intake in this study sample are consistent with intake data from national samples: sweet snacks was 14.9% kcal in the current study, which is comparable to the added sugar consumption of all-race adolescents as reported in the U.S. National Center for Health Statistics (NCHS) data 2005–2008 [31]; the daily caloric intake from SSB consumption was 157.93 kcal, comparable to the 2011–2014 NCHS data where the average SSB intake among AA boys was 167 kcal, and girls was 156 kcal [14]. Moreover, consistent with the NCHS data, the result of this study also suggests an increased risk of SSB intake among older adolescents [14]. This is probably related to the increasing autonomy, independence, and opportunities to decide what to drink outside the house among older adolescents [11].

An inverse association between self-efficacy and sweet snacks and SSB consumption was found in this study; adolescents with higher healthy diet self-efficacy are less likely to consume sweet snacks and SSB consumption. This result aligns with another study conducted among U.S. multi-racial adolescents [32]. According to the SCT, higher confidence in performing a healthy behavior result in a higher probability of actually engaging in that behavior [33]. The results of this study reinforce the benefit of increasing self-efficacy of a healthy diet to decrease sweet snacks and SSB consumption in AA adolescents living in low-income neighborhoods.

The results of this study also found that higher healthy diet intention was associated with lower sweet snacks and SSB consumption in adolescents. While the findings from the current study demonstrate potential relationships, they are consistent with an intervention study in U.S. adolescents, which concludes that adolescents who receive intention training consume less SSB [34]. Further, the result of this study is also supported by a systematic review that found higher sugary snacking and SSB consumption among adolescents with a higher intention of unhealthy diet [35].

The results of this study indicated no significant association between healthy diet knowledge and consumption of sweet snacks and SSB. This result is aligned with other studies that report that knowledge of health risk was not associated with SSB consumption among adolescents [36]. Another study conducted in London, UK also showed no significant relationship between children’s nutritional knowledge and their sweet snacks consumption [37]. According to SCT, knowledge is a more distal construct from behavior [33]; a lack of significant relationship between knowledge and health behavior is supported by the theory that knowledge by itself is insufficient to elicit a behavior [38].

The results of this study found a positive association between healthy diet outcome expectancies with SSB, but not with sweet snacks consumption. According to a systematic review on psychosocial factors of children and adolescents’ eating behavior, the associations of outcome expectancies and dietary behavior are not consistent across studies [35]. Evidence on the association between outcome expectancies and sweet snacks and SSB consumption in U.S. adolescents is limited. However, a study on Taiwanese adolescents found that adolescents with greater expectations of negative outcomes consume fewer SSB [39], although this study involves vocational students majoring in food and beverages who on average had good nutritional knowledge and negative attitude toward SSB consumption and my not directly apply to the population in this study.

The current study found no association between sweet snacks and SSB consumption and BMI-for-age. In contrast to our findings, a trial showed that a decrease in SSB consumption resulted in lower BMI among American adolescents [40]. The difference in the result may be due to the difference in the diet measurement methods; the respondents in the current study were not trained to quantify consumption, while the study used semi-quantitative FFQ to measure the intake. Furthermore, the relationships between sweet snacks and SSB consumption with BMI-for-age in this study are potentially confounded by physical activity. In addition, this study implemented an observational design rather than an environmentally controlled trial.

One strength of this study includes the focus on adolescence, a key period of development. While the national data shows consistent increases in obesity prevalence over the time, limited studies focus on obesity risk factors in this population, particularly among AA adolescents with low income. Furthermore, adolescence is an ideal target for obesity interventions as they experience higher autonomy in food decision-making. Thus, understanding the psychosocial factors associated with sweet snacks and SSB consumption is important to develop behavioral interventions to improve adolescents’ diet. This study also has limitations. First, the use of a cross-sectional design prevents us from understanding the causal relationships of variables studied. Second, the sweet snacks and SSB consumption are self-reported, which may be subject to recall bias and influenced by social desirability. However, we expected this bias resulted in non-differential misclassification as the subjects of this study have homogenous characteristics. Third, we used a semi-quantitative FFQ to measure the intake, so the results depend on what items were included on the list of sweet snacks and SSB. Finally, the Cronbach’s alphas for the food intention questionnaire were weak, indicating poor inter-relatedness between questions. However, the questionnaire was a modified version of a questionnaire which was previously validated in AA adolescents in Baltimore [21].

## 5. Conclusions

Social cognitive theory is often used to explain sugar consumption behavior [41,42]. The current study builds upon evidence suggesting that higher healthy diet self-efficacy and intentions are associated with lower consumption of sweet snacks and SSB in AA adolescents living in low-income neighborhoods. However, no associations between knowledge of sweet snacks and SSB consumption were found. These findings suggest the importance of improving adolescents’ self-efficacy and food intention to reduce sweet snacks and SSB consumption. Further, the results suggest that designing an intervention that aims solely on improving knowledge may be insufficient. Future interventions using a longitudinal design should be conducted to explore the causal and dose-response relationship of self-efficacy, food intention, and outcome expectancies with sweet snacks and SSB consumption in adolescents.

## Figures and Tables

**Table 1:** Baseline characteristics of adolescents and caregivers in the B’More Healthy Community for Kids (BHCK) trial (2013–2014).

Characteristics	*n* (%)
**Adolescent characteristics**	
**Sex**	
Female	237 (45.77)
Male	200 (54.23)
**Adolescent age (years)**	
9	6 (1.37)
10	100 (22.88)
11	113 (25.86)
12	80 (18.31)
13	70 (16.02)
14	62 (14.19)
15	6 (1.37)
**Weight status (BMI-for-Age)**	
Normal	224 (51.38)
Overweight	98 (22.48)
Obese	114 (26.15)
**Caregiver characteristics**	
**Sex**	
Female	382 (87.61)
Male	54 (12.39)
**Caregiver age** Mean (SD)	38.23 (10.22)
**Caregiver education level**	
Less than high school	73 (16.78)
High school or GED	170 (39.08)
Some college or associates	124 (28.51)
Bachelor’s or graduate school	35 (8.05)
Vocational school or others	33 (7.59)
**Household income ($/year)**	
0–10,000	100 (22.94)
10,001–20,000	95 (21.79)
20,001–30,000	81 (18.58)
>30,000	160 (36.70)
**Total caloric intake (kcal)** Mean (SD)	1735.96 (1063.82)
**Total caloric intake from sugar (kcal)** Mean (SD)	130.64 (88.37)
**Total sweet snacks consumption (% from daily caloric intake)**	14.93 (7.28)
**Total sugar sweetened beverages consumption (g)** Mean (SD)	363.52 (367.11)
**Total sugar sweetened beverages consumption (kcal)** Mean (SD)	157.93 (157.98)

SD = standard deviation; g = grams; kcal = kilocalories; BMI = Body Mass Index; GED = General Educational Development; SSB = Sugar Sweetened Beverages.

**Table 2:** The pattern of sweet snacks consumption and sugar sweetened beverages (SSB) based on sociodemographic factors among African American adolescents aged 9–15 (*n* = 437) in the B’More Healthy Community for Kids (BHCK) trial at baseline (2013–2014).

Characteristics	Total Sweet Snacks Consumption (% Total Daily Kcal Intake)		Total Sugar Sweetened Beverages Consumption (g)		Total Sugar Sweetened Beverages Consumption (kcal)	
	Mean (SD)	*p*-Value	Mean (SD)	*p*-Value	Mean (SD)	*p*-Value
**Adolescent characteristics**						
**Sex**						
Female	14.37 (0.46)	0.07	375.06 (24.89)	0.54	165.93 (10.76)	0.18
Male	15.60 (0.53)		349.84 (24.56)		148.48 (10.48)	
**Adolescent age**						
9–12	15.32 (0.42)	0.10	314.84 (19.10)	<0.001 **	138.61 (8.29)	<0.001**
13–15	14.11 (0.61)		469.01 (35.63)		199.81 (15.24)	
**Weight status (BMI-for-age)**						
Normal	15.33 (0.49)	Ref	397.46 (24.49)	Ref	173.88 (10.53)	Ref
Overweight	15.26 (0.88)	0.94	315.57 (44.39)	0.19	134.85 (19.09)	0.10
Obese	13.94 (0.49)	0.09	339.69 (42.17)	0.54	147.19 (18.13)	0.49
**Caregiver characteristics**						
**Sex**						
Female	15.13 (0.37)	0.11	362.75 (18.86)	0.89	157.63 (8.13)	0.84
Male	13.42 (1.00)		364.76 (49.23)		158.09 (20.86)	
**Caregiver education level**						
Less than high school	12.62 (0.84)	Ref	356.88 (42.85)	Ref	153.80 (18.44)	Ref
High school or GED	14.85 (1.01)	0.03 *	409.08 (50.63)	0.61	177.99 (22.05)	0.76
Some college or associates	16.37 (1.06)	<0.001 **	355.95 (53.42)	0.99	154.20 (23.24)	0.84
Bachelor’s or graduate school	14.21 (1.48)	0.29	236.99 (74.39)	0.11	104.18 (32.39)	0.12
Vocational school or others	15.53 (1.51)	0.06	296.96 (76.64)	0.62	130.77 (33.05)	0.55
**Household income ($/year)**						
0–10,000	14.18 (0.73)	Ref	393.52 (36.70)	Ref	170.53 (15.80)	Ref
10,001–20,000	15.08 (1.04)	0.39	319.46 (52.58)	0.64	139.34 (22.64)	0.58
20,001–30,000	15.54 (1.09)	0.21	410.79 (54.86)	0.78	176.67 (23.62)	0.78
>30,000	14.98 (0.93)	0.39	345.58 (46.78)	0.55	150.93 (20.14)	0.54

SD = standard deviation; g = grams; kcal = kilocalories; Ref = reference; BMI = Body Mass Index; GED = General Educational Development, * *p*-value < 0.001, ** *p*-value < 0.001.

**Table 3:** Multiple linear regression results of sweet snacks and sugar sweetened beverages (SSB) consumption on psychosocial factors among African American adolescents aged 9–15 (*n* = 437) in the B’More Healthy Community for Kids (BHCK) trial at baseline (2013–2014).

Characteristics	Total Sweet Snacks Consumption (% kcal of Total Daily kcal Intake)		Total Sugar Sweetened Beverages (SSB) Consumption (kcal)	
	OR (95% CI) Unadjusted	OR (95% CI) Adjusted ^1^	OR (95% CI) Unadjusted	OR (95% CI) Adjusted ^1^
Food-related knowledge	1.05 (0.78–1.43)	1.19 (0.85–1.68)	1.02 (0.98–1.73)	1.01 (0.96–1.05)
Outcome expectancies	0.92 (0.79–1.07)	0.93 (0.80–1.08)	0.98 (0.96–0.99) *	1.02 (0.97–1.06)
Self-efficacy	0.84 (0.74–0.95) **	0.81 (0.71–0.93) *	0.98 (0.96–0.99) *	0.99 (0.97–1.01)
Food intentions	0.47 (0.34–0.66) **	0.43 (0.30–0.61) **	0.91 (0.87–0.95) **	0.92 (0.88–0.97) **

CI = Confidence Interval; OR = Odds Ratio; kcal = kilocalories; * *p*-value <0.05; ** *p*-value <0.001; **^1^** Adjusted for adolescent age and sex; caregiver age, sex, education level; household income, intervention zone, SNAP participation.

**Table 4:** Multiple logistic regression results of weight status (BMI-for-age) on sweet snacks and sugar sweetened beverages (SSB) consumption among African American adolescents aged 9–15 in B’More Healthy Community for Kids (BHCK) trial at baseline (2013–2014).

Sweet Snacks and SSB Consumption	Normal vs. Overweight/Obese		Normal/Overweight vs. Obese		Normal/Overweight/Obese vs. >Obese	
	OR (95% CI) Unadjusted	OR (95% CI) Adjusted ^4^	OR (95% CI) Unadjusted	OR (95% CI) Adjusted ^4^	OR (95% CI) Unadjusted	OR (95% CI) Adjusted ^4^
Total sugary foods consumption ^1^	1.01 (0.98–1.04)	1.01 (0.98–1.04)	1.02 (0.99–1.06)	1.03 (0.99–1.06)	1.15 (0.82–1.62)	1.13 (0.82–1.55)
Total SSB consumption (g) ^2^	1.14 (0.92–1.40)	1.00 (0.78–1.29)	1.03 (0.81–1.31)	0.85 (0.63–1.14)	1.57 (0.19–12.7)	0.94 (0.07–12.70)
Total SSB consumption (kcal) ^3^	1.17 (0.94–1.45)	1.05 (0.81–1.36)	1.03 (0.81–1.32)	0.85 (0.63–1.16)	1.70 (0.19–15.18)	1.09 (0.07–17.25)

CI = Confidence Interval; OR = Odds Ratio; ^1^ Measured in percentage of total kcal from sweets; ^2^ Measured in grams consumed per day; ^3^ Measured in kcal consumed per day; ^4^ Adjusted for adolescent age, sex, total daily caloric intake.

## Data Availability

The data presented in this study are available on request from the corresponding author. The data are not publicly available due to ethical issues.
